# A Novel Reconstruction Method for Measurement Data Based on MTLS Algorithm

**DOI:** 10.3390/s20226449

**Published:** 2020-11-12

**Authors:** Tianqi Gu, Chenjie Hu, Dawei Tang, Tianzhi Luo

**Affiliations:** 1 School of Mechanical Engineering and Automation, Fuzhou University, Fuzhou 350108, China; tqgu2014@fzu.edu.cn (T.G.); n180227022@fzu.edu.cn (C.H.); 2 Centre for Precision Technologies, University of Huddersfield, Huddersfield HD1 3DH, UK; d.tang@hud.ac.uk; 3 CAS Key Laboratory of Mechanical Behaviour and Design of Materials, Department of Modern Mechanics, University of Science and Technology of China, Hefei 230022, China

**Keywords:** Moving Least Squares, surface profile, outliers, reconstruction method

## Abstract

Reconstruction methods for discrete data, such as the Moving Least Squares (MLS) and Moving Total Least Squares (MTLS), have made a great many achievements with the progress of modern industrial technology. Although the MLS and MTLS have good approximation accuracy, neither of these two approaches are robust model reconstruction methods and the outliers in the data cannot be processed effectively as the construction principle results in distorted local approximation. This paper proposes an improved method that is called the Moving Total Least Trimmed Squares (MTLTS) to achieve more accurate and robust estimations. By applying the Total Least Trimmed Squares (TLTS) method to the orthogonal construction way in the proposed MTLTS, the outliers as well as the random errors of all variables that exist in the measurement data can be effectively suppressed. The results of the numerical simulation and measurement experiment show that the proposed algorithm is superior to the MTLS and MLS method from the perspective of robustness and accuracy.

## 1. Introduction

Nowadays, benefitting from the development of reverse engineering and computer technology, the meshless method widely used for reconstructing the discrete data has been studied by varieties of scholars, and consequently different types of meshless methods have been proposed [1,2]. Among all the numerical methods, the meshless method obtains the local approximants of the entire parameter domain only based on the nodal points instead of elements [3,4]. In view of its outstanding features, it has replaced traditional estimation methods in some research fields [5,6]. The meshless methods that have been widely used include the Moving Least Squares (MLS), the smoothed particle hydrodynamics, the radial basis function, etc., in which the MLS method is one of the most popular methods [7].

After years of development, the MLS method has already been employed to solve engineering and scientific problems in many fields [8,9,10]. For example, Dabboura et al., used the MLS method to acquire the result of the Kuramoto–Sivashinsky equation [11]. Amirfakhrian and Mafikandi applied the MLS method to approximate the parametric curves to verify the reliability [7]. Lee [12] proposed an improved moving least-squares algorithm to approximate a set of unorganized points with a smooth curve without self-intersections, in which Euclidean minimum spanning tree, region expansion and refining iteration were used. Then, Belytschko et al. [13] first presented the Element-Free Galerkin (EFG) method by combining the weak form of the Galerkin and MLS method, which can obtain the approximation function by using only the nodes and has been applied to fracture and elasticity problems. The advantages of the EFG method are to avoid the problem of volumetric locking and improve the convergence rate and stability compared with the Finite Element Method (FEM). This method is still an important method in the current application of MLS method. For example, the EFG method was employed in solving the Signorini boundary value problems by Li et al. [14]. The difficulty of nonlinear inequality restrictions is solved by a projection iterative scheme. Compared to the FEM, this scheme improves the convergence rate as well as calculation accuracy. After the EFG method, the MLS based meshless method has been developed rapidly, deriving many classic methods [15,16], for example, the meshless local Petrov–Galerkin method [7], the improved element-free Galerkin method [17], the moving least square reproducing kernel method [18], and the direct meshless local Petrov-Galerkin method [19].

Although the MLS has been widely used due to its good fitting features [20,21], it also has its own drawbacks, especially in approximating curves and surfaces. As a reconstruction method, the local coefficients are obtained by least squares (LS) method which assumes that the error exists in the dependent variable of the measurement data [22,23]. To take the random errors of all variables into account [24,25], Scitovski et al. [26] generalized the traditional algorithms based on the work of Lancaster et al. and put forward the Moving Total Least Squares (MTLS) method. According to data processing and error theory, the MTLS method is logical to handle the errors-in-variables (EV) model. Nevertheless, neither of these two approaches is a robust method for discrete data reconstruction [10,27]. Even though there is only one outlier in the discrete data, the accuracy of the reconstruction will be seriously affected. In the practical application of 3D digital model reconstruction, the data is always associated with errors that are generated in both data collection and processing [28]. Therefore, the reconstruction results have different degrees of deviation varying with the degrees of data pollution.

Since the occurrence of outliers in the data is inevitable, it is critical to define and process them to control the negative effects on geometric model reconstruction so that the fitted data can better reflect the true information [29]. There are two common approaches for handling outliers. One approach is to set a threshold to remove the data with larger values, which may lead to the loss of true information [30]. The other is to correct them by changing the weights of the outliers, which brings the risk of different degrees of contamination. Specifically, the threshold in the former approach, obtained by probability and statistics [31], directly determines whether the reconstructed model can reflect the geometric feature information of the true object. Therefore, the selection of threshold must be reasonable, which is actually hard to achieve under different conditions. In the latter approach, adding small weights to outliers in the measurement data is difficult to achieve as well because how to exactly predict the generated impact of the method will be an issue [32].

To suppress the influence of outliers, we propose an improved reconstruction algorithm called the Moving Total Least Trimmed Squares (MTLTS) method for accurate curve and surface profile analysis, in which the TLTS method using Singular Value Decomposition (SVD) [33] is employed to deal with the abnormal data in the influence domain. The remainder of the paper is structured as follows: in the Section 2, a description of the MTLS and MLS method is drawn briefly; the Section 3 introduces the principle of the MTLTS method; in the Section 4, we give the results of numerical simulation and measurement experiment and make a brief analysis; lastly, we show the conclusions in the Section 5.

## 2. The Introduction of the Basic Theory

### 2.1. MLS Method

The conventional LS method considered as a global method, especially in the field of coordinate metrology, has been an actual standard for curve fitting. However, it cannot express the local feature information accurately when the model is complicated. Compared with the LS method, the MLS method is similar to the piecewise method, in which each estimated point domain corresponds to a segmentation. If the local data is sufficient to reflect the true feature information, the MLS method has good local approximation property and the ability of low order fitting [34].

Consider **θ** {**θ**_1_, **θ**_2_, …, **θ***_N_*} and **ϑ**{**ϑ**_1_, **ϑ**_2_, …, **ϑ***_N_*} are nodes in a bounded region Ω in space *R^D^* [35]. The approximation function *f_h_* for each point **θ** in the MLS method is defined as

(1) $$f_{h}\left(\theta \right)=\sum_{j=1}^{m}b_{j}\left(\theta \right)a_{j}\left(\theta \right)=b^{T}\left(\theta \right)a\left(\theta \right)$$

where **a**(**θ**) = [**a**_1_(**θ**), **a**_2_(**θ**), …, **a***_m_*(**θ**)]*^T^* is a vector of unknown coefficient **a***_j_*(**θ**), (*j* = 1, 2, …, *m*), **b**(**θ**) is a vector of the basis **b***_j_*(**θ**), and all of their dimension is *m*. In view of the low order fitting characteristics, we only consider the most commonly used linear least squares estimation. In this article, the basis functions of curve and surface reconstruction are **b** = [1, **θ**]*^T^* and **b** = [1, **θ**, **ϑ**]*^T^* respectively.

To obtain the unknown optimal parameter vector **a**(**θ**), the MLS method solves it by determining the minimum sum of absolute differences of all nodes between *f*(**θ**) and *f_h_*(**θ**) function [36]. We know that the error function is based on Equation (1), in which the independent variable is **θ**. The function is given as

(2) $$\begin{array}{ll} J & =\sum_{I=1}^{n}w\left(|\theta -\theta_{I}|/r\right){\left[f_{h}\left(\theta \right)-f\left(\theta_{I}\right)\right]}^{2}\\ & =\sum_{I=1}^{n}w\left(|\theta -\theta_{I}|/r\right){\left[\sum_{j=1}^{m}b_{j}\left(\theta_{I}\right)a_{j}\left(\theta \right)-f\left(\theta_{I}\right)\right]}^{2}\\ & ={\left(Ba-f\right)}^{T}W(Ba-f) \end{array}$$

where

$$\begin{array}{l} W=diag(w_{1}(s),\;w_{2}(s),\;\cdots ,\;w_{n}(s))\\ f={\left[f(\theta_{1}),\;f(\theta_{2}),\;\cdots ,\;f(\theta_{n})\right]}^{T}\\ B=\left[\begin{array}{cccc} b_{1}(\theta_{1}) & b_{2}(\theta_{1}) & \cdots & b_{m}(\theta_{1})\\ b_{1}(\theta_{2}) & b_{2}(\theta_{2}) & \cdots & b_{m}(\theta_{2})\\ \vdots & \vdots & \ddots & \vdots \\ b_{1}(\theta_{n}) & b_{2}(\theta_{n}) & \cdots & b_{m}(\theta_{n}) \end{array}\right] \end{array}$$

and *s* = |(**θ**−**θ***_I_*)|/*r*. *r* represents the radius of the influence domain. The weight function *w*(*s*) is used to ensure a good local approximation. The value of *w*(*s*) has decreasing property with the distance between the fitting point and the nodal points and makes sure that the value of the fitting point will be affected only by these points in the influence domain. Many types of functions can meet this requirement [37], so the selection of the weight function is not fixed but determined by the accuracy under the conditions of continuity and smoothness. This article adopts the following function

(3) $$w(s)=\left\{ \begin{array}{ll} 1-6s^{2}+8s^{3}-3s^{4} & \left|s\right|\leq 1\\ 0 & \left|s\right|>1 \end{array}\right.$$

**Figure 1:**
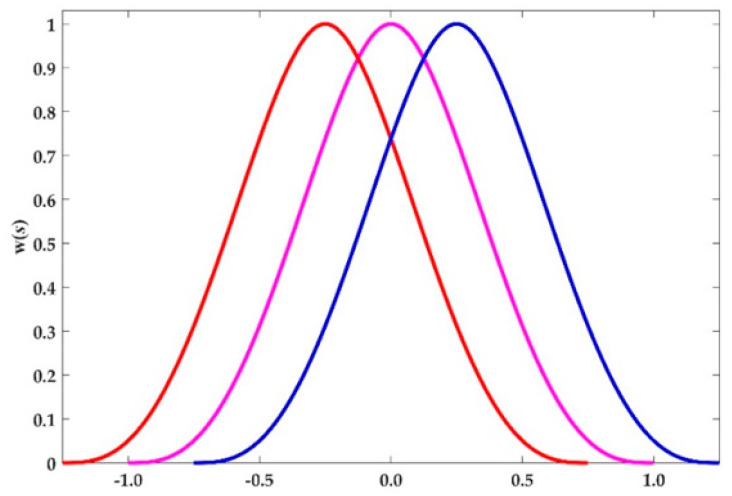
The compact support of weight function.

This weight function is shown in Figure 1.

**Figure 2:**
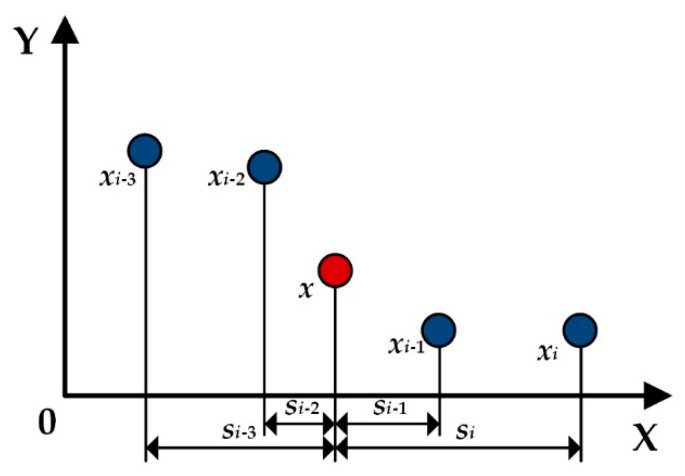
The definition way of weight function in the influence domain.

The weight function plays an important role in the local approximants, which provides weight values for the points in the influence domain, as shown in Figure 2.

The weight of each point in the influence domain can be determined by the projection distance from this point to nodal point, which ensures that the approximation is globally continuous and the shape functions satisfy the compatibility condition.

Obtain the partial derivative of Equation (2) and make it equal 0, i.e.,

$$\frac{\partial J}{\partial a}=B^{T}WBa-B^{T}Wf=0$$

The sum of errors of MLS approximation function gets extreme value. Then, the optimal coefficient vector is obtained

(4) $$a\left(\theta \right)={Μ}^{-1}\left(\theta \right)L\left(\theta \right)f$$

where

$$M(\theta )=B^{T}WB\;,\;L(\theta )=B^{T}W$$

Substitute Equation (4) into Equation (1), it obtains

(5) $$f_{h}\left(\theta \right)=b^{T}\left(\theta \right)a\left(\theta \right)=b^{T}\left(\theta \right)M^{-1}\left(\theta \right)L\left(\theta \right)f$$

### 2.2. MTLS Method

The MLS method gains the local approximation coefficients by using the least squares estimation, which considers that the model of the random error is the Gauss–Markov (GM) model. Then, R. Scitovski et al. [26] proposed the MTLS method to process the random errors that exist in all variables.

Suppose that (*x_j_*, *y_j_*), *j* = 1, …, *n* is a set of data in the curve *y* = *f*(*x*). When errors occur to all the variables of measurement data, gain the local approximation parameters (*c*_0_, *c*_1_) ∈*R* of the function

(6) $$y_{j}=f(x_{j}+\delta_{j})+\varepsilon_{j}$$

in the sense of TLS estimation. Unlike the MLS method, the MTLS method gains the coefficients through determining the minimum sum of weighted squared orthogonal distances. In the actual measured data, random errors always exist in both dependent and independent variables of data. According to the error theory, the MTLS method is more logical to process the EV model than the MLS method [28,38].

## 3. Proposed MTLTS Method

### 3.1. LTS Method

A brief introduction is given to the LTS method of the polynomial model in this part. Get a set of data (*x_j_*, *y_j_*), *j* = 1, ..., *n* in the curve *y* = *f*(*x*). Then, we can express its model [39] as

(7) $$Y=X\beta_{t}+e$$

where **X** is a *n* × *p* matrix, **β***_t_* (**β***_t_* ∈ **β**, *t* = 1, 2, …, $C_{n}^{P}$) is a *p* × 1 regression coefficient vector and **e** is the error matrix. For each parameter vector **β***_t_*, the residual vector is defined as **r***_t_* = **Y**−**Xβ***_t_*. The unknown **r***_t_* is a n-dimensional vector whose square is defined in ascending order as **$r_{t}^{2}$** = [$r_{1}^{2}$, $r_{2}^{2}$, …, $r_{n}^{2}$], (0 ≤ $r_{1}^{2}$≤…≤$r_{j}^{2}$≤…≤ $r_{n}^{2}$). Rousseeuw [40] first introduced LTS estimator, and its expression is defined as follows

(8) $$\beta_{LTS}=\underset{\beta_{t}\in \beta }{\arg\min}\sum_{j=1}^{h}r_{j}^{2}$$

where the value of the trimming constant *h*∈ (*n*/2, *n*) depends on the degree of data pollution [41]. In the calculation, the integers h equals (*n* + *p* + 1)/2 and *P* = *p* + 1. It is known to us that the breakdown point is the most basic standard to judge whether an estimator is robust enough or not. When *h* = *n*/2, the breakdown point of the LTS estimator is up to 1/2. Especially, when *h* is equal to *n*, it corresponds to the least squares estimation and its breakdown point is close to zero [42]. This means that the modelling process can automatically eliminate (*n*−*h*) larger residuals as long as the percentage of data pollution is no more than 50% [43].

### 3.2. MTLTS Method

**Figure 3:**
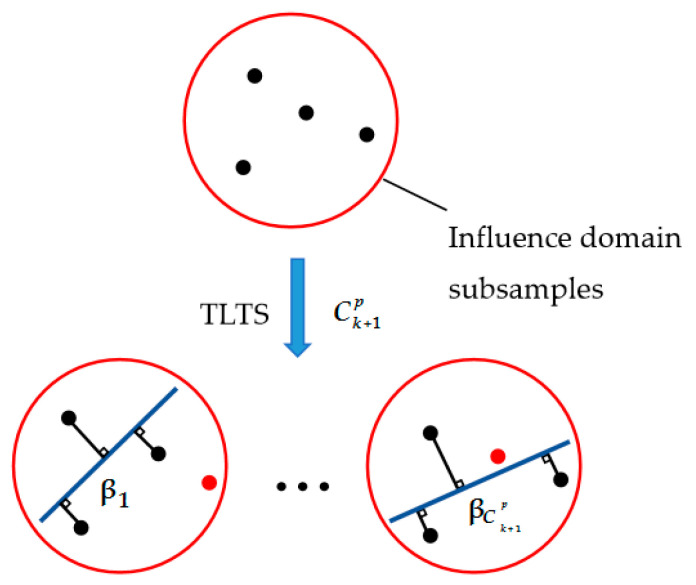
Schematic graph of the Moving Total Least Trimmed Squares (MTLTS) method.

As stated above, the MTLS method is susceptible to the outliers, but it considers the random errors that exist in all variables. Even though the LTS method is robust, it cannot express the local geometry feature information of the complicated model. Therefore, we propose a MTLTS method, in which the TLTS method (a combination of the TLS and LTS) is employed to acquire the fitting coefficients of influence domain (Figure 3).

For the proposed algorithm, the TLTS method is employed to determine the local optimal parameter vector in the influence domain. Let *k* + 1 < *n*, where n and *k* + 1 are the numbers of the nodes in the whole parameter domain and in the influence domain, respectively. For an arbitrary influence domain, there are $C_{k+1}^{P}$ subsamples based on the TLTS method.

For each subsample, the SVD based TLS method is utilized for obtaining the regression coefficients **β***_t_* (**β***_t_* ∈ **β**, *t* = 1, 2, …, $C_{k+1}^{P}$). The function model is defined as

(9) $$\begin{array}{ll} AX=B\\ A=\;A_{1}+\Delta A\; & B=B_{1}+\Delta B \end{array}$$

where **A**_1_ and **B**_1_ are the true values, **A** and **B** represent the actual measured values, and the errors between them are ∆**A** and ∆**B**.

An augmented matrix **C** is made for the subsample and the SVD of **C** is described by

(10) $$C\;:=W\left[A\;\;\;\;B\right]=U\Sigma V^{T}$$

where **W** = diag(*w*(**x**−**x**_1_), *w*(**x**−**x**_2_), …, *w*(**x**−**x***_P_*)) is the weight matrix, the right part singular matrix **V** = [**V**_1_, **V**_2_, …, **V***_P_*_+1_], **V***_P_*_+1_ = [*v*_1, *P*+1_, *v*_2, *P*+1_, …, *v_P_*_+1, *P*+1_]*^T^*, and the singular matrix **Σ** = diag(*σ*_1_, *σ*_2_, …, *σ_P_*_+1_).

If *σ_P_* ≠ *σ_P_*_+1_, the solution of TLS is unique, it can be gained by the following formula [24,25]

(11) $$\beta_{t}=\frac{-1}{v_{P+1,P+1}}\left[\begin{array}{l} v_{1,P+1}\\ v_{2,P+1}\\ \;\vdots \\ v_{P,P+1} \end{array}\right]$$

The squared residuals can be obtained by the local coefficient vector and defined in ascending order as $r_{t}^{2}$ = [$d_{1}^{2}$, $d_{2}^{2}$, …, $d_{j}^{2}$, …, $d_{P}^{2}$], (0 ≤ $d_{1}^{2}$ ≤ … ≤ $d_{j}^{2}$ ≤ … ≤$d_{P}^{2}$). The TLTS method is different from the traditional LTS method as it takes into account the random errors that exist in all variables, in which the distance $d_{j}^{2}$(*j* ∈ [1, 2, …, *P*]) is the squared residual in the orthogonal direction. On this occasion, the sum of the squared residual of the smallest h-subset of each subsample is defined as

(12) $$S_{t}=\sum_{j=1}^{h}d_{j}^{2}$$

The coefficient matrix **β** = [**β**_1_, **β**_2_, …, **β**_$C_{k+1}^{P}$_] can be obtained by repeating calculations. The TLTS estimation is used to determine the corresponding optimal coefficient vector by finding the smallest *h*-subset. The estimation is defined as

(13) $$\beta_{TLTS}=\underset{\beta_{t}\in \beta }{\arg\min}S=\underset{\beta_{t}\in \beta }{\arg\min}\left\{S_{1},S_{2},\ldots \;,S_{t},\ldots \;,S_{C_{k+1}^{P}}\right\}$$

Move the fixed point throughout the domain and repeat the previous steps, in which the estimation for each point is independent. Then, we get the reconstructed curve or surface. In this paper, we set *h* = [(*k* + *p* + 2)/2].

## 4. Case Study

To validate the data fitting performance of the MTLTS method, numerical simulations as well as experimental examples are given in this section. In the numerical simulation, the tested data is simulated by artificially adding random errors and outliers. The spline weight function introduced is applied to all cases.

### 4.1. Case 1

Take the function

(14) $$y=1.1(1-x+2x^{2})e^{\frac{-x^{2}}{4.5}}\;$$

as an example. A uniformly distributed set of nodes (*x_j_*, *y_j_*) from the Equation (14) is first selected. Then, get the data (*x_jm_*, *y_jm_*) by adding outliers (0, Δ*y_i_*) and the random errors (*δ_j_*, *ɛ_j_*) to (*x_j_*, *y_j_*), where the random errors obey the normal distribution with a mean value of zero.

The sum of absolute differences between the fitting points and the theoretical points

(15) $$s=\sum_{j=1}^{n}\left|y_{j}-y_{jn}\right|$$

is employed in evaluating their performance where *y_jn_* and *y_j_* are the fitting points and theoretical points.

**Figure 4:**
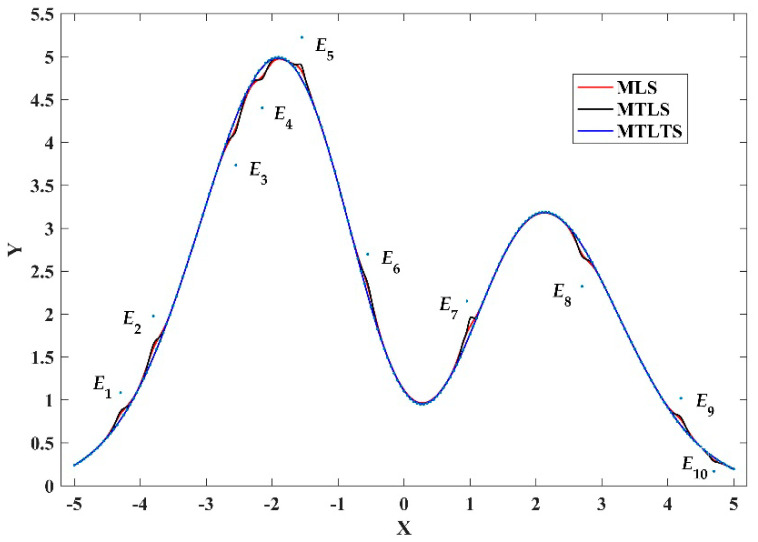
The fitting curves of three methods in Case 1.

Let *n* = 201 and *r* = (*x_jm_*(201) − *x_jm_*(1)) × 3/100 in Case 1, in which *x_jm_*(1) = −5 and *x_jm_*(201) = 5. Figure 4 presents the fitting curves obtained by the MLS, MTLS and MTLTS. The summation of the differences for these methods under different conditions are shown in Table 1, respectively. These points marked in Figure 4 are outliers. In the cases of this paper, we provided relatively more outliers in the whole domain to verify the proposed algorithm.

The fitting accuracy also can be evaluated by the Root Mean Square (*RMS*) value. The results are still consistent with the sum of absolute differences, as shown in Table 2. In order to avoid repetition, the *RMS* values are not placed in the other cases.

### 4.2. Case 2

**Figure 5:**
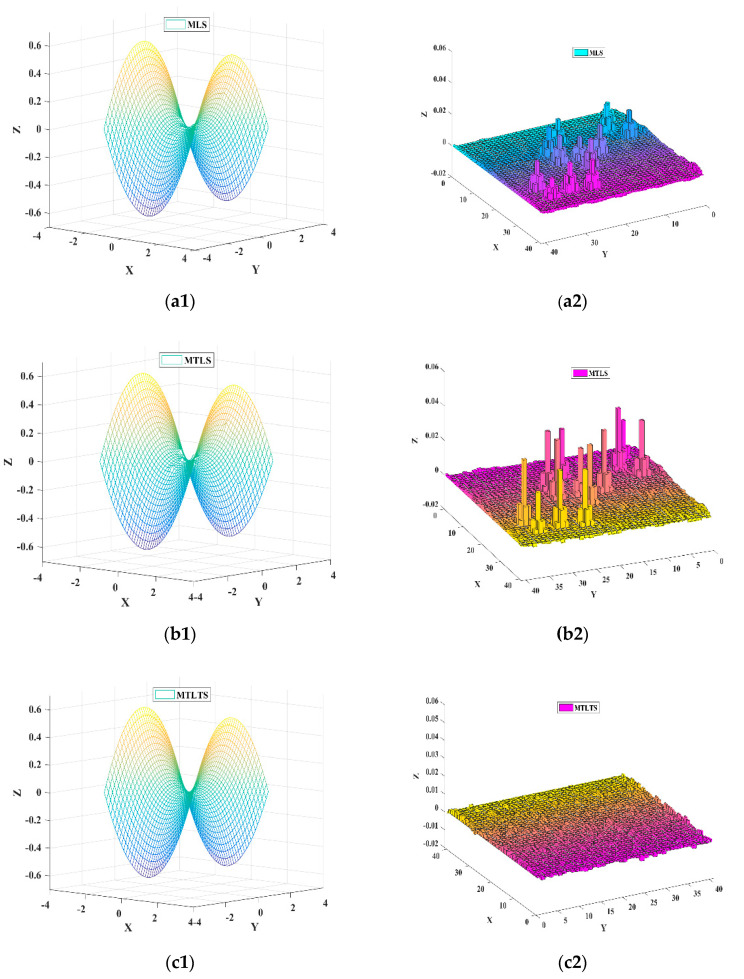
The fitting and error surfaces of three methods in Case 2. (**a1**) The fitting surface of MLS; (**a2**) The error surface of MLS; (**b1**) The fitting surface of MTLS; (**b2**) The error surface of MTLS; (**c1**) The fitting surface of MTLTS; (**c2**) The error surface of MTLTS.

Take the function

(16) $$z=(x^{2}-y^{2})/10$$

and define the square area Ω = [−2.4, 2.4] × [−2.4, 2.4] as the definition domain of this case. Let *n* = 1681 and *r* = (*x_jm_*(41) + *y_jm_*(41)) × 7/100 in Case 2, in which *x_jm_*(41) = 2.4 and *y_jm_*(41) = 2.4. The surface reconstruction is evaluated by using

(17) $$s=\sum_{j=1}^{n}\left|z_{j}-z_{jn}\right|$$

where *z_jn_* and *z_j_* are fitting points and theoretical points. Following the same approach described in Case 1, the fitting results of three methods under different random conditions are shown in Table 3 and Figure 5, respectively.

**Figure 6:**
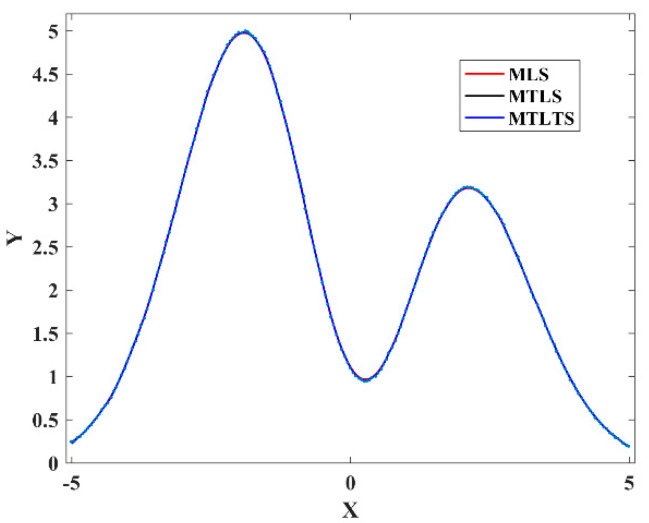
Fitting curves obtained by the Moving Least Squares (MLS), Moving Total Least Squares (MTLS), and MTLTS.

**Figure 7:**
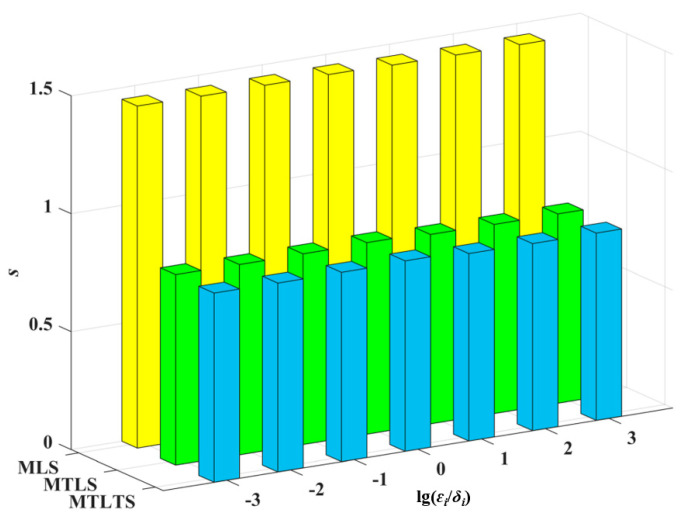
The trend of the *s* values.

From Figure 4 and Figure 5, we know that MLS and MTLS are not robust model reconstruction methods. For these two methods, outliers have a great influence on the estimation of nearby fitting points and even lead to distortion of the results. In comparison, the sum of differences of the MTLTS method is much smaller in the presence of the contaminated data. To validate the fitting accuracy of the MTLTS method when there are no abnormal points in the discrete data, we still take the curve function to get the data in contrast to Case 1. As shown in Figure 6, the curves reconstructed by the three methods provide good approximation characteristics. However, the comparison of the result listed in Table 4 and Figure 7 shows that the fitting differences of MTLTS method are obviously lower than the other two methods.

To obtain the corresponding CPU-times amongst IMTLS, MLS, and MTLS, the Case I is taken as an example and MATLAB is used to test the computation load of these algorithms. All procedures are conducted on a PC with Intel(R) Core^TM^ i7 2.7/2.9 GHz 8 RAM (Santa Clara County, CA, USA). The results are shown in Table 5.

### 4.3. Case 3

**Figure 8:**
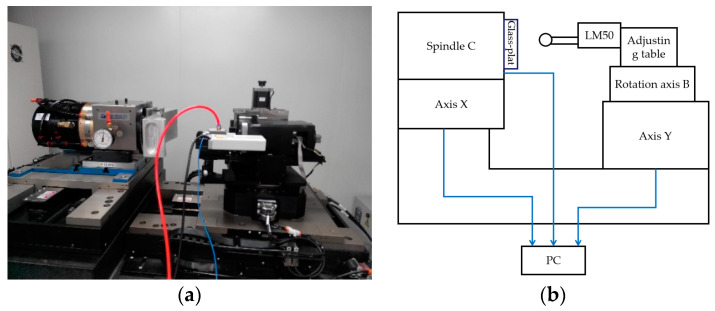
Precision measurement platform. (**a**) Measurement platform; (**b**) Schematic diagram.

To further verify the performance of MTLTS method, it is also applied to fit the measurement data obtained by a precision measurement platform, as shown in Figure 8.

**Figure 9:**
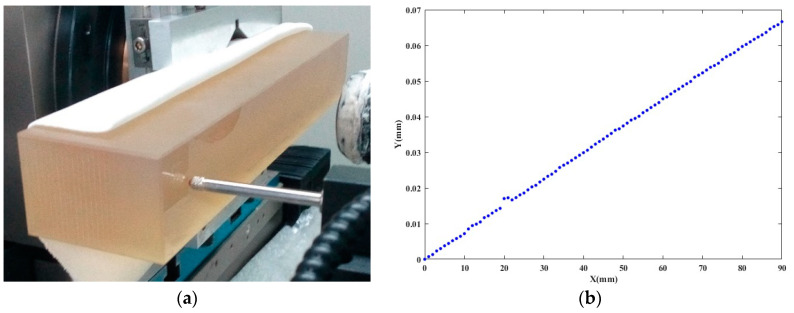
The measurement experiment of optical flat. (**a**) Measurement for optical plat; (**b**) Measurement data of optical plat.

The measurement system is based on the LM50 laser-interferometric gauging probe and performs measurement of the surface profile of the processed workpiece. The employed point-contact ruby probe has a low contact pressure while offering a high measurement accuracy. At the planned layout point, the surface profile data of the workpiece is obtained by the **X**-axis and LM50, respectively. **X**-axis has a repetitive positioning error of about 41 nm and the sensor has a repetitive error of around 127 nm. As shown in Figure 9, the measurement data was obtained experimentally by measuring the profile of an optical flat, which has a peak-to-valley (PV) value of 31 nm. 

**Figure 10:**
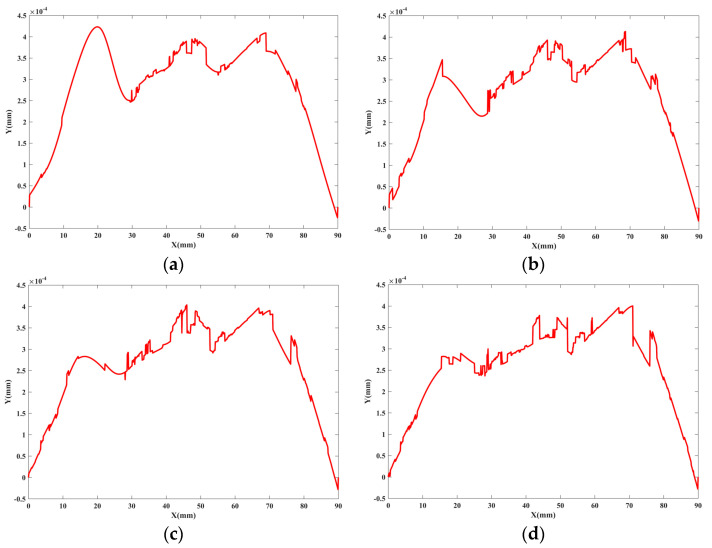
The fitting curves of MTLTS method. (**a**) MTLTS ($C_{k+1}^{k}$); (**b**) MTLTS ($C_{k+1}^{k-1}$); (**c**) MTLTS ($C_{k+1}^{k-2}$); (**d**) MTLTS ($C_{k+1}^{k-3}$).

The measurement length is 90 mm and the total number of sampling points is 91. MLS, MTLS, and MTLTS method are applied to process the experimental data and TLTS method is used for linear regression. Then, the corresponding straightness values are used to verify the performances of these methods. The fitting results of the MTLTS with different $C_{k+1}^{P}$ parameters are shown in Figure 10. The straightness values obtained by the three reconstruction methods are listed in Table 6.

**Figure 11:**
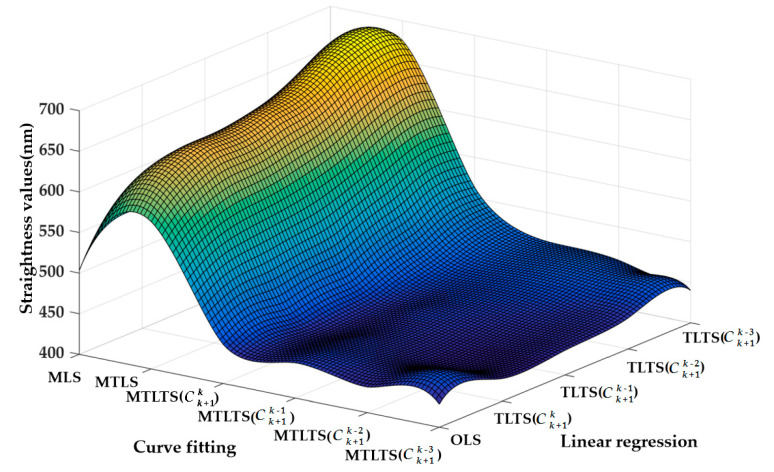
The trend of the straightness value.

As shown in Table 6, MLS, MTLS, and MTLTS with different $C_{k+1}^{P}$ parameters are applied to fit the measurement data of the optical plat, and TLS and TLTS with different $C_{k+1}^{P}$ parameters are used for linear regression. Figure 11 shows the variation trend of the straightness values when different curve fitting and linear regression methods are chosen.

As shown in Figure 11, MLS and MTLS method are both greatly influenced by the outliers. With the increase of P value of TLTS method, the results of evaluated straightness get worse, which also illustrates the robustness of TLTS method for linear regression. In comparison, the obtained straightness of MTLTS method is always closest to the standard value. Furthermore, with the increase of P value of MTLTS method (i.e., with the decrease of nodes for determining the local approximate coefficients within a single influence domain), the results of MTLTS method tend to be stable, which confirms the effectiveness of the proposed method.

**Figure 12:**
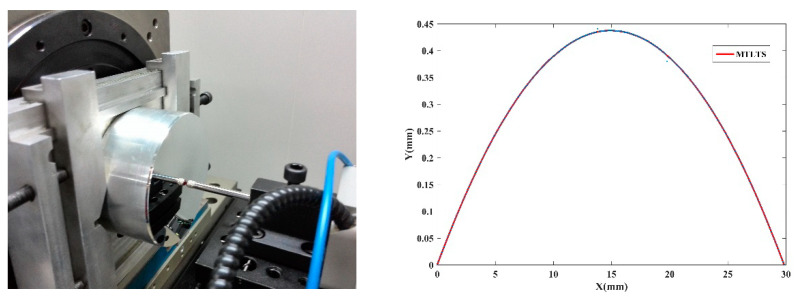
The measurement of the generatrix of spherical surface.

The same measuring instrument is used to measure the generatrix of spherical surface, as shown in Figure 12.

**Figure 13:**
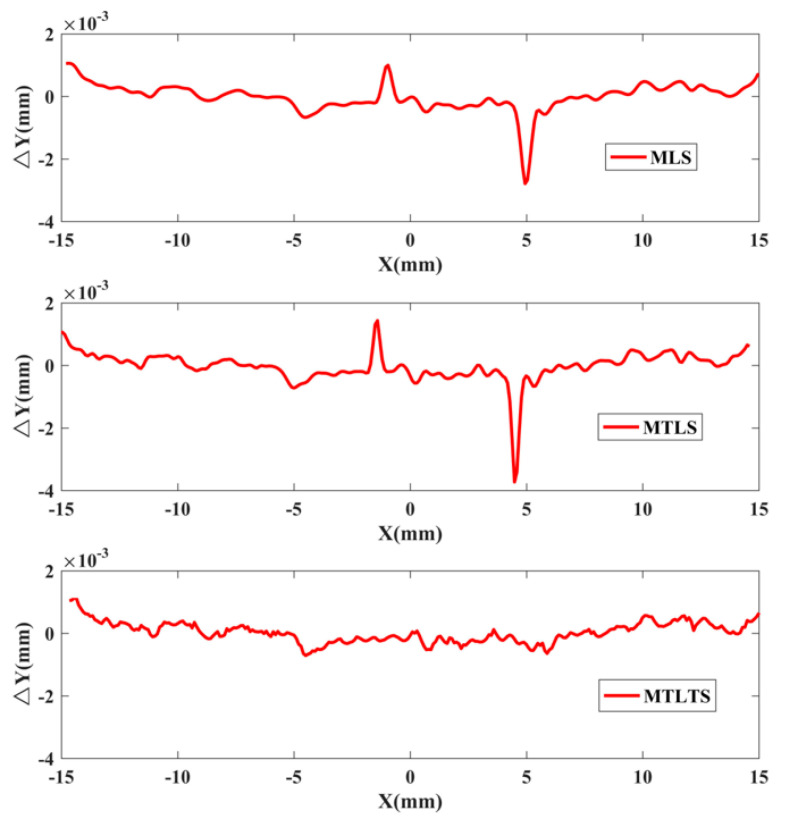
The error graphs processed by three methods.

The radius of the spherical surface is 254.0677 mm tested by Taylor Hobson PGI 1240 profilometer. The profile data are fitted by MLS, MTLS, and MTLTS respectively. The reconstructed data is processed for circular registration by the simulated annealing algorithm. Figure 13 shows the error graphs and the PV values of the three methods are obtained in Table 7.

**Figure 14:**
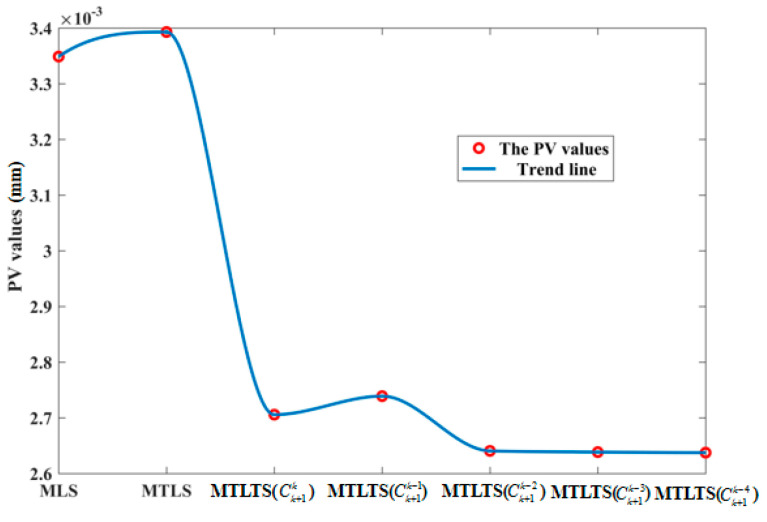
The PV value trend graph.

As shown in Table 7, the PV value processed by the MTLTS method is significantly smaller than the other two methods. In order to verify the stability of the algorithm when different numbers of points of influence domain are eliminated, Figure 14 shows the PV value trend graph of the process. As the P value increases, the PV values gradually become stabilized.

The proposed MTLTS algorithm has combined the advantages of the MTLS and LTS method and involves outstanding characteristics. Although the measurement data has outliers, it is still able to reconstruct the curve or surface from the discrete data with high accuracy by applying the improved method. Furthermore, the comparison with another two numerical estimation methods represents that the accuracy and robustness of the MTLTS algorithm have been significantly enhanced whether there are outliers in the data or not.

## 5. Conclusions

In this study, a robust reconstruction algorithm for measurement data, based on the MTLS method, is presented by introducing the TLTS method to the influence domain for finding an optimal local parameter vector. We studied the algorithm from the perspective of calculation and theory. Owing to the construction principle of the algorithm, it does not only possess the property of acquiring the shape function with high order continuity and consistency under the basis function with low order. In addition, the robust algorithm overcomes the shortcoming of lacking robustness that is difficult to be solved for the traditional numerical estimation methods (MLS and MTLS). To verify the proposed method in terms of fitting performance, all three methods are employed for fitting the data generated by numerical simulation and experimental measurement. The results show that the MTLTS method has significant advantages over the MTLS and MLS method whether there are outliers or not, which proves the performance of this robust algorithm.

## Figures and Tables

**Table 1:** The fitting results by three methods in Case 1.

Variance		*s*		
*δ_j_*	*ɛ_j_*	MLS	MTLS	MTLTS
0.000001	0.001	5.361290	4.942031	0.830636
0.00001	0.001	5.360357	4.941088	0.829239
0.0001	0.001	5.360257	4.940890	0.829474
0.001	0.001	5.364536	4.951253	0.844504
0.001	0.0001	5.361439	4.944982	0.838307
0.001	0.00001	5.363066	4.947494	0.838965
0.001	0.000001	5.362450	4.947406	0.839584

**Table 2:** The fitting results by three methods in Case 1.

Variance		*RMS*		
*δ_j_*	*ɛ_j_*	MLS	MTLS	MTLTS
0.000001	0.001	0.041089	0.046397	0.005262
0.00001	0.001	0.041040	0.046296	0.005187
0.0001	0.001	0.041022	0.046294	0.005258
0.001	0.001	0.041020	0.046289	0.005256
0.001	0.0001	0.041011	0.045915	0.005300
0.001	0.00001	0.040943	0.046178	0.005124
0.001	0.000001	0.041071	0.046482	0.005138

**Table 3:** The fitting results by three methods in Case 2.

Variance		*s*		
*δ_j_*	*ɛ_j_*	MLS	MTLS	MTLTS
0.000001	0.001	2.380559	2.595104	0.959207
0.00001	0.001	2.383201	2.601161	0.960295
0.0001	0.001	2.385662	2.602565	0.959230
0.001	0.001	2.449716	2.695886	1.025807
0.001	0.0001	2.269477	2.373187	0.724637
0.001	0.00001	2.274174	2.556756	0.735747
0.001	0.000001	2.264168	2.360344	0.721755

**Table 4:** The results of three methods for the data without outliers.

Variance		*s*		
*δ_j_*	*ɛ_j_*	MLS	MTLS	MTLTS
0.000001	0.001	1.447804	0.802516	0.793830
0.00001	0.001	1.447193	0.801830	0.792997
0.0001	0.001	1.448305	0.803013	0.794529
0.001	0.001	1.452026	0.811992	0.807419
0.001	0.0001	1.450294	0.808405	0.802493
0.001	0.00001	1.448076	0.806652	0.800710
0.001	0.000001	1.449720	0.807843	0.801968

**Table 5:** The corresponding CPU-times amongst MTLTS, MLS, and MTLS for Case I.

Number of the Points		The CPU-Times (s)	
	MLS	MTLS	MTLTS
201	0.1560	0.0156	0.9516
501	1.5288	0.0312	2.7300
1001	5.2728	0.0780	3.2136
2001	16.0369	0.2496	5.0388

**Table 6:** The straightness values of three methods (nm).

	Curve Fitting	MLS	MTLS	MTLTS ($C_{k+1}^{k}$)	MTLTS ($C_{k+1}^{k-1}$)	MTLTS ($C_{k+1}^{k-2}$)	MTLTS ($C_{k+1}^{k-3}$)
Linear Regression							
TLS		503	581	448	443	431	428
TLTS ($C_{k+1}^{k}$)		549	633	459	442	428	427
TLTS ($C_{k+1}^{k-1}$)		558	642	469	445	434	430
TLTS ($C_{k+1}^{k-2}$)		586	670	496	453	451	437
TLTS ($C_{k+1}^{k-3}$)		591	675	513	464	459	439

**Table 7:** The peak-to-valley (PV) values of three methods.

Methods	PV Values (nm)
MLS	3348
MTLS	3392
MTLTS	2705
